# Evaluation of Nanomagnetite–Biochar Composite for BTA Removal

**DOI:** 10.3390/nano15020115

**Published:** 2025-01-14

**Authors:** Carolina Guida, Nathaniel Findling, Valérie Magnin, Fabienne Favre Boivin, Laurent Charlet

**Affiliations:** 1 ISTerre, University Grenoble Alpes, University Savoie Mont Blanc, CNRS, IRD, University Gustave Eiffel, 38058 Grenoble, France; carolina.guida.m@gmail.com (C.G.); nathaniel.findling@universite-paris-saclay.fr (N.F.); valerie.magnin@univ-grenoble-alpes.fr (V.M.); 2 Institute des Technologies de l’Environnement Construit, University of Applied Sciences and Arts Western Switzerland, 1700 Fribourg, Switzerland; fabienne.favre@hefr.ch

**Keywords:** biochar, nanomagnetite, benzotriazole, adsorption, composite

## Abstract

In this study, the removal of benzotriazole (BTA), a pervasive aquatic contaminant widely used for its anti-corrosion, UV-stabilizing, and antioxidant properties, by nanomagnetite, biochar, and nanomagnetite–biochar composite is investigated. Nanomagnetite and nanomagnetite–biochar composite were synthesized under anoxic conditions and tested for BTA removal efficiency at neutral pH under both oxic and anoxic conditions at different time scales. Within the short time scale (up to 8 h), the removal of BTA by nanomagnetite–biochar composite was shown to be due to BTA deprotonation by the nanomagnetite surface. Through proton liberation, Fe²⁺ is released in accordance with the reaction Fe₃O₄ + 2H⁺ → Fe₂O₃ + Fe²⁺ + H₂O, which likely influences BTA complexation and its possible redox degradation. On the longer time scale, biochar achieved higher removal efficiency: 50% BTA removed within 48 h, due to formation of a ternary complex with surface Ca^2+^ ions, or 75% BTA removed after HCl biochar acid wash followed by Ca^2+^ surface saturation. As BTA presents significant environmental risks due to its extensive industrial applications, the present study offers critical insights into the mechanisms of BTA removal by nanomagnetite–biochar composite, and highlights the potential of such materials for water treatment applications.

## 1. Introduction

Access to clean water to the growing world population is the sixth of the seventeen sustainable development goals identified by the United Nations [1]. Clean water is vital for environmental sustainability and human health, but it faces increasing threats from contaminants. These contaminants include traditional pollutants such as heavy metals and pesticides, as well as emerging substances like pharmaceuticals and personal care products [2]. While the presence of these pollutants in the environment is not new, advancements in detection technology have revealed their widespread occurrence in water sources, groundwater, and even treated drinking water [3]. This heightened awareness highlights the urgent need for effective strategies to safeguard water quality for both current and future generations.

Nanoscale magnetite (Fe₃O₄) has attracted considerable interest as a highly efficient adsorbent for water treatment, owing to its distinct physicochemical properties. Consisting of both Fe^2^⁺ and Fe^3^⁺ ions, magnetic nanoadsorbents provide various benefits, such as superior adsorption performance, rapid recovery due to their high specific surface area, ease of manipulation, and relatively low production costs [4]. In addition, their magnetic nature allows for easy separation and facilitates repeated use and regeneration through simple processes, positioning them as a sustainable option for the removal of pollutants. However, while these nanoparticles are a promising treatment option, they are not yet mature enough for large-scale water treatment applications [5]. One challenge is the clogging of filtration pores due to the small particle size, which limits their practical deployment. To overcome this issue, it is important to develop a reliable, cost-effective, scalable, and efficient method for the synthesis of physically stable magnetic nanoparticles. Such advancements will be of great benefit for both the practical implementation of these materials and the progression of fundamental research in the field.

As climate change concerns and fossil fuel scrutiny intensify, the quest for sustainable, biobased materials has surged [6]. Biochar, especially in its modified forms, has emerged as a promising solution for various environmental applications [7]. Its carbon-negative production process helps mitigate the greenhouse effect and aligns with dual carbon policy requirements [8]. Additionally, biochar is more cost-effective than activated carbon, costing 5 USD /kg compared to 5.6 USD/kg [9]. Producing 1 kg of biochar uses 6.1 MJ of energy, while activated carbon requires 97 MJ, making biochar suitable for large-scale use as an adsorbent. Its diverse applications include enhancing soil fertility, promoting microbial activity, and adsorbing pollutants such as heavy metals and organic contaminants [10,11]. The adsorption efficiency of biochar is largely determined by its chemical and physical properties, which are influenced by its manufacturing process and biomass source [12,13].

Benzotriazole (BTA) is a widely used corrosion inhibitor found in various industrial processes as aircraft deicing and anti-icing fluids, household products, and pharmaceuticals. Recent studies have revealed significant residual levels of BTA in various environmental matrices, including plants, fish, air, human urine, and surface and groundwater, raising critical concerns regarding their ecological and health impacts [14]. BTA has become a significant environmental contaminant due to its resistance to conventional wastewater treatments and its stability and bioaccumulative properties in aquatic environments. Despite its generally low acute toxicity, BTA has sublethal impacts—such as endocrine disruption, hepatotoxicity, neurotoxicity, and the promotion of endometrial carcinoma development—underscoring the urgent need for effective and affordable remediation techniques that avoid creating additional toxic byproducts and that ensure safe and straightforward disposal.

This study investigates on the adsorption efficiency and mechanism of nanomagnetite (M) and commercially sold pyrolyzed oak wood biochar (B) for the removal of BTA. We also propose a novel modification of this biochar by coating it with magnetite nanoparticles (MB) to enhance its sorption properties. This modification is particularly interesting as most existing work on MB composites has reported high removal rates for different organic pollutants [15,16,17,18], positioning it as a viable solution for mitigating BTA contamination in aquatic environments. The magnetic properties of the modified biochar facilitate its easy recovery from environmental matrices, while the coating of biochar particles helps prevent pore clogging in water treatment filters, offering a practical and scalable approach for large-scale water treatment applications. Here, the physicochemical properties of magnetite, biochar, and biochar–magnetite composite were characterized using the XRD, FTIR, BET, and SEM techniques. Additionally, their capacity and mechanism for BTA uptake were investigated through a kinetic approach.

## 2. Materials and Methods

### 2.1. Materials

Benzotriazole (CAS No. 95-14-7, 99% purity) was sourced from ReagentPlus^®^, Merck KGaA, Darmstadt, Germany. Calcium chloride (99% purity) was purchased from Sigma-Aldrich, Merck KGaA, Darmstadt, Allemagne and sodium hydroxide (99% purity) from Carl Roth GmbH + Co. KG, Karlsruhe, Germany. Hydrochloric acid (48%, Extra Pure, SLR grade) was obtained from Fisher Chemical. For the synthesis of nanomagnetite, iron(II) chloride tetrahydrate and iron(III) chloride hexahydrate (both with 99% purity) were procured from Sigma-Aldrich, Merck KGaA, Darmstadt, Allemagne. Biochar (Japanese Binchotan) was acquired from Takesumi company, Viet-Nam.

### 2.2. Adsorbed Materials Treatment

Biochar (B): B was initially crushed using a hammer and mortar to reduce the particle size. The resulting powder was then sieved to obtain particles with a size range between 100 µm and 250 µm.

Washed biochar (WB): For the preparation of both WB and a magnetite/biochar composite (MB), after sieving, the biochar was immersed in a 1 M HCl solution for 48 h at room temperature to remove ash and other surface contaminants. After the acid treatment, the biochar was thoroughly rinsed with ultrapure water in four successive washes to ensure the complete removal of residual acid and any remaining impurities. Following this washing step, the biochar was placed in an N_2_-filled glove box and left to dry overnight.

Additionally, to detect the presence of Ca^2+^ in the biochar matrix, two batch experiments were conducted. In the first experiment biochar was added to ultrapure water at a concentration of 10 g/L. In the second experiment, the pH of the suspension was adjusted to 3 using hydrochloric acid (HCl). Both experiments were maintained for 48 h to reach equilibrium. After equilibration, the suspension was analyzed for calcium concentration using ICP-AES.

Nanomagnetite (M) and MB composite synthesis: M and MB composite were synthesized using the co-precipitation method, involving aqueous solutions of Fe^3+^, Fe^2+^, and WB. All procedures were conducted in a glove box under an N_2_ atmosphere at room temperature to maintain an inert environment, and all solutions were prepared using N_2_-degassed water to prevent oxidation prior to mineral synthesis. For the M synthesis, a mixed ferrous/ferric chloride solution (Fe^2+^/Fe^3+^ ratio of 0.5) was prepared with 25 mL FeCl_2_ at 0.8 M and 25 mL FeCl_3_ at 1.6 M concentrations. Then, 60 mL of 3 M NH_4_OH solution was slowly added to the Fe^2+^/Fe^3+^ mix under continuous stirring. The resulting suspension was stirred for 24 h, followed by washing the precipitate with degassed water. This washing process was repeated six times, replacing the supernatant each time. The final product was conserved in 0.1 mmol NaCl.

For the MB composite, the same procedure was followed, but with the addition of 4.63 g of WB to the mixed ferrous/ferric chloride solution. This resulted in a composite containing 50 wt% biochar, with the remaining steps identical to the magnetite synthesis.

### 2.3. Material Characterization

All materials, including B, WB, MB, and magnetite, underwent thorough characterization using several analytical techniques: powder X-ray diffraction (XRD), Brunauer–Emmett–Teller (BET) surface area analysis, Fourier-transform infrared spectroscopy (FTIR), and scanning electron microscopy (SEM).

BET: The specific surface area (SSA) of the samples was measured using the Brunauer–Emmett–Teller (BET) adsorption method at 77 K with nitrogen gas. This analysis was performed using a Belsorp Max (Bel Japan) volumetric gas sorption analyzer. For the analysis, powder of B, magnetite, and MB were loaded into a glass sample cell within a glovebox and subsequently dried under vacuum at 80 °C for 12 h. The SSA was calculated using the BET equation over the relative pressure (P/P₀) range of 0 to 0.4.

SEM: SEM imaging was carried out using a scanning electron microscope operating at 16 kV at ISTerre in Grenoble, France. The sample was prepared by carefully applying a small amount of material onto the surface of electrically conductive carbon double-sided tape, which was affixed to the sample mount. Both backscattered electrons (BSE) and secondary electrons (SE) detection modes were employed to capture detailed images of the sample’s morphology.

TEM: TEM was utilized to examine the nanomagnetite samples, offering detailed insights into their morphology, elemental composition, and structural features. For sample preparation, a small quantity of each solid was dispersed in ethanol preconditioned under an inert atmosphere in a glovebox. The dispersion underwent brief ultrasonic treatment outside the glovebox to achieve uniform particle redistribution before being returned to the glovebox for dilution. Subsequently, the diluted solutions were carefully deposited onto 200-mesh pure carbon copper TEM grids (TED PELLA, INC.) via drop-casting. To preserve sample integrity, the grids were transferred to the TEM under anoxic conditions, minimizing air exposure during mounting. Analytical measurements were performed using a JEOL 2100F TEM at the IMPMC, Sorbonne University, Paris under optimized operating conditions to reduce sample degradation and ensure accurate characterization.

FTIR: FTIR spectroscopy was utilized to identify functional groups present in the three samples. The measurements were performed in the spectral range of 4000 to 400 cm^−1^ using a Nicolet 6700 spectrophotometer equipped with a Thermo Fisher Scientific diamond crystal. The resolution was set to 4 cm^−1^, and each spectrum was obtained by averaging 64 scans for both the sample and the background.

XRD: X-ray powder diffraction was conducted using a Bruker D8 diffractometer equipped with CuKα radiation (λ = 1.54 Å). The diffractometer was configured in a Debye–Scherrer arrangement, utilizing an elliptical mirror to produce a high-flux, parallel incident beam. A SolX Si (Li) solid-state detector was used to collect the diffracted X-rays. The diffraction patterns were recorded with a step size of 0.026° over a 2θ range of 0° to 60° at ambient temperature. To prevent oxidation, the XRD samples were loaded inside a glovebox into an airtight polymethyl methacrylate sample holder, capped with a transparent dome.

### 2.4. Kinetic Studies

Kinetic experiments were performed in duplicate using degassed, deionized water at room temperature (~25 °C) and a neutral pH of 6.8. The BTA initial concentration was set at 100 µM and the solid concentration was 2 g/L for both raw (B) and acid-washed biochar (WB), as well as with nanomagnetite (M) and magnetite/biochar composite (MB). To prevent contamination, the experiments were conducted in hermetically sealed glass bottles. These bottles were wrapped in aluminum foil and paper to exclude light. At predetermined intervals, ranging from 0 to 2 weeks, 5 mL samples were withdrawn using sterile syringes. The samples were immediately filtered through a 0.45 µm membrane to remove particulates prior to BTA analysis.

Additional experiments with WB were carried out under identical conditions, this time adding Ca^2+^ concentrations of 0.125 mM and 0.25 mM, to assess the effect of divalent cations on the adsorption process. Two other experiments were conducted within a controlled atmosphere glove box to investigate the interactions between M and MB composite at a solid concentration of 15 g/L. These experiments were carried out under a nitrogen atmosphere to prevent the oxidation of nanomagnetite.

### 2.5. Kinetic Modeling

The adsorption kinetics were investigated using two widely recognized models: the pseudo-first-order (PFO) and pseudo-second-order (PSO) models. These models were employed to describe the rate at which adsorption occurred and to estimate the adsorption capacity at equilibrium.

Variables and definitions:

- t (hours): Time at equilibrium.
- qt (mg/g): Amount of adsorbate adsorbed per unit mass of adsorbent at time t.
- qe (mg/g): Adsorption capacity at equilibrium.
- kn (g/mg·h or h−1): Rate constant for a reaction of order n.
- C0 (mg/L): Initial concentration of adsorbate in the solution.
- Ce (mg/L): Concentration of adsorbate in the solution at equilibrium.
- mads (g): Mass of the adsorbent used in the experiment.
- Vsol (L): Volume of the solution containing the adsorbate.

Experimental calculation of q_t_:

The amount adsorbed at time t, *q*_*t*_, was calculated using the following equation:

(1) $$q_{t}=\frac{C_{0}-C_{e}}{m_{ads}}.V_{sol}$$

Model evaluation using sum of squared errors (SSE):

The accuracy of the kinetic models was assessed by calculating the sum of squared errors (SSE) between the experimental q_t_ values and those predicted by the models:

(2) $$SSE=\overset{n}{\underset{i=1}{\sum }}{\left[q_{t,cal}-q_{t,exp}\right]}_{i}^{2}$$

where *n* represents the number of data points.

Pseudo-first-order model (PFO):

The PFO model describes the adsorption rate using the following differential equation:

(3) $$\frac{dq_{t}}{dt}=k_{1}\left(q_{e}-q_{t}\right)$$

This can be integrated to obtain the non-linear form:

(4) $$q_{t}=q_{e}\left(1-e^{-k_{1}*t}\right)$$

The half-life of the reaction (time required for qt to reach half of q_e_) is given by

(5) $$t_{1/2}=\frac{ln2}{k_{1}}$$

Pseudo-second-order model (PSO):

The PSO model assumes that the adsorption rate is proportional to the square of the difference between q_e_ and q_t_:

(6) $$\frac{dq_{t}}{dt}=k_{2}{\left(q_{e}-q_{t}\right)}^{2}$$

The corresponding non-linear form is

(7) $$q_{t}=\frac{q_{e}^{2}\cdot k_{2}\cdot t}{1+q_{e}\cdot k_{2}\cdot t}$$

The initial adsorption rate, h_0_, for the PSO model is

(8) $$h_{0}=q_{e}^{2}\cdot k_{2}$$

### 2.6. Wet Chemistry

UV–vis: The BTA concentrations in solution samples were analyzed at 260 for B and WB and 275 nm for magnetite and MB with an Agilent Cary 300 UV–vis spectrophotometer. The DLs for BTA were 1 µM for 260 nm and 0.6 µM for 275 nm. The QLs for 260 nm and 275 nm were 3.1 and 1.9 μM, respectively.

ICP−AES: Total Ca concentrations in selected samples were determined by ICP−AES (Varian 720 ES) at a wavelength of 318 nm. Ca Roth standard with 1000 ppm was used to prepare an external calibration with a range of 0.1−100 ppm. Analytical precision (RSD) and recovery (CMS) were determined from replicate analyses (*n* = 2). The RSDs calculated for the 10 ppm standard were 3% for Ca. The CMS was 98%. The detection limit (DL) for Ca was 10.4 μM, and the quantification limit (QL) was 31.5 μM.

## 3. Results and Discussion

### 3.1. Nanomagnetite and Biochar Image Characterization

**Figure 1:**
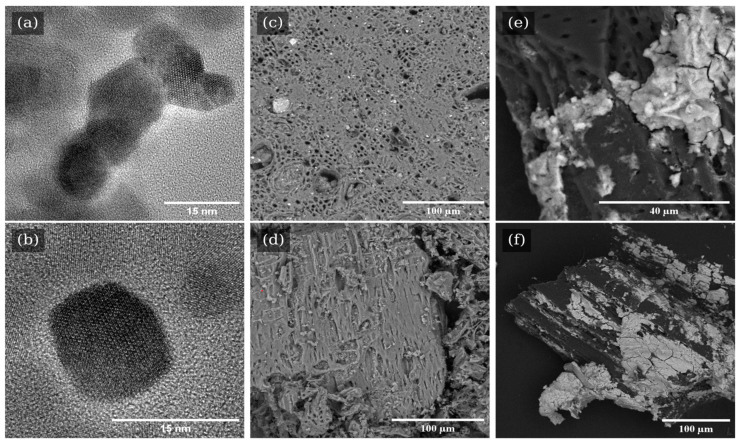
TEM bright field images of nanomagnetites (**a**,**b**) and SEM images of biochar samples: unwashed biochar (**c**) and biochar washed with HCl (**d**), and nanomagnetite–biochar composite (**e**,**f**). Images (**c**,**d**) were captured using backscattered electron imaging, while images (**e**,**f**) were obtained in secondary electron mode.

TEM images of the M samples reveal that the nanomagnetite consists of well-ordered domains, each less than 15 nm in size, as shown in Figure 1a,b. The consistent domain structure observed across multiple samples indicates reproducibility and stability in the synthesis procedure. SEM imaging of raw oak wood biochar material depicts well-defined vessels structure, with approximately 2 μm diameter (Figure 1c,d). The white rectangular particles exceeding 50 μm in length were attributed to calcite crystals. Indeed, biochar treated with hydrochloric acid (or washed WB) was free from these large calcite crystals and other ash remains (Figure 1d). Such treatment enhanced the porosity and improved the pore homogeneity and size within the biochar, leading to a more microporous, and thus reactive, material.

### 3.2. Crystalline and Surface Properties

**Figure 2:**
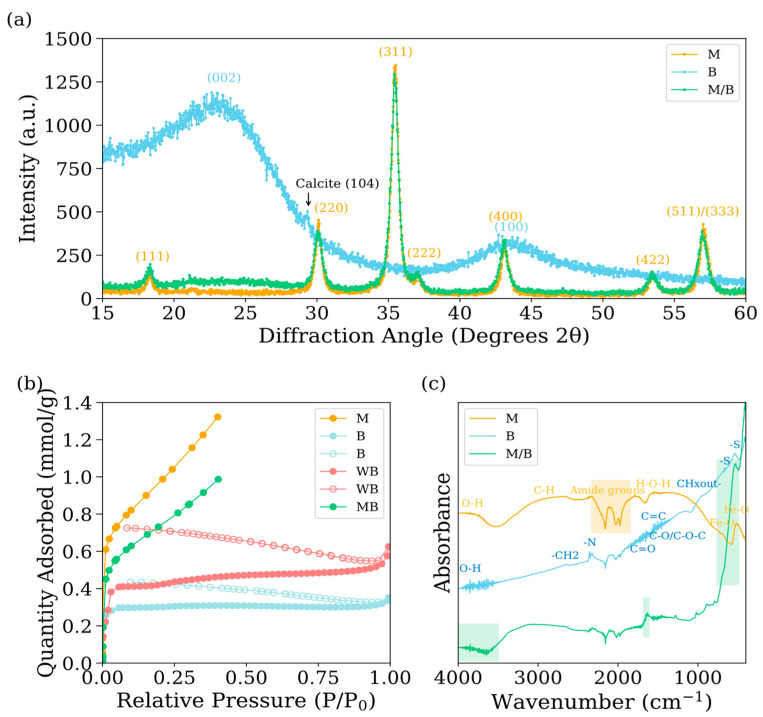
(**a**) X-ray diffraction patterns of nanomagnetite (yellow), biochar (blue), nanomagnetite-biochar composite (green), and acid washed biochar (red) (**b**). BET adsorption isotherms for specific surface area calculation and (**c**) FTIR spectra of nanomagnetite, biochar, and nanomagnetite–biochar composite. XRD and FTIR data use the same color symbols as the BET results. For BET, empty dots indicate nitrogen desorption isotherms. The XRD references used for the characterization of the different materials correspond to JCPDS PDF 00-019-0629 for magnetite and ICDD PDF-2 #41-1487 (graphite) for biochar in B and MB materials.

Further structural analysis through XRD confirmed the presence of magnetite cubic structures (18.328°, 30.054°, 35.41°, 35.074°, 43.184°, 53.402°, and 56.964°) and graphite (peaks at 23.044° and 43.688°) within the materials. A minor peak observed at 29.336° in B sample suggests the formation of calcite crystals, likely resulting from the commercial final stages of production, during which the biochar was cooled using clay and ash (Figure 2a). The Brunauer–Emmett–Teller (BET) adsorption isotherms revealed distinct characteristics among the materials under investigation (Figure 2b). Both nanomagnetite (M) and the nanomagnetite–biochar composite (MB) displayed Type II isotherms, which are associated with multilayer adsorption processes characteristic of mesopores ranging from 2 to 50 nm. Biochar (B) exhibited a Type I isotherm, indicative of the presence of micropores with diameters less than 2 nm, corroborating the macroporosity identified through SEM images. The BET-specific surface area (SSA) measurements were determined to be 79 m^2^/g for M, 28 m^2^/g for B and 59 m^2^/g for MB. These findings are consistent with, yet distinct from, the existing literature, which reports SSA values for this specific biochar ranging from 0.183 m^2^/g [19] to 270 m^2^/g [20]. Such variability in SSA is likely attributable to differences in experimental conditions, particularly particle size screening. It is well-established that finer particles typically exhibit larger SSA [21]. Despite this variability, the findings from both the literature and the present study suggest that biochar is characterized by limited microporosity and a predominance of macropores.

### 3.3. FTIR Analysis

The FTIR analysis of the materials reveals distinct functional groups indicative of their chemical composition and surface characteristics. For M sample, bands at 3820 cm^−1^ and 2925 cm^−1^ indicate the presence of O-H and C-H groups, respectively. Amide groups are detected between 2330 and 1846 cm^−1^. A band at 1559 cm^−1^ corresponds to H-O-H vibrations, while Fe-O vibrations are confirmed by bands at 656 and 497 cm^−1^ [22].

For B, the sample exhibited distinct characteristics indicative of lignocellulosic-based biochar [23]. The bands between 3849 and 3625 cm^−1^ correspond to hydroxyl groups (-OH), characteristic of hydroxylated surfaces [24]. Aliphatic and aromatic CH₂ groups are identified by the band at 2660 cm^−1^, while nitrile (-N) groups are evidenced by bands in the range of 2350 to 1980 cm^−1^ [25]. The bands at 1650 cm^−1^ signify the presence of carbonyl groups (C=O), while aromatic C=C bonds and carboxyl group stretching are marked by bands at 1583–1384 cm^−1^ [26]. Surface functional groups such as C–O/C–O–C appear at 1160 cm^−1^, while aromatic CHx out-of-plane deformation is evident at 945 cm^−1^ [27].

For the WB composite, the FTIR spectra indicate the presence of surface groups from both B and M, with notable differences arising from nanomagnetite crystal growth using B as a nucleation agent. The increased intensity of the OH and CH bands at approximately 4000 and 3000 cm^−1^ in the WB composites suggests the formation of iron hydroxides on the B surface. The Fe-O bands, originally observed at 497 and 656 cm^−1^, shift to 518 and 694 cm^−1^, respectively, which suggests there is an interaction between the carbonaceous materials and the magnetite nanoparticles [28]. A new aromatic C=C band appears at 1630 cm^−1^, reflecting polymerization and carbonization of the biochar during the synthesis of WB composites [24,25]. These changes emphasize the synergistic effects between B and M during composite synthesis, leading to enhanced functional properties.

### 3.4. UV–Vis Spectroscopy Insights into BTA Adsorption

**Figure 3:**
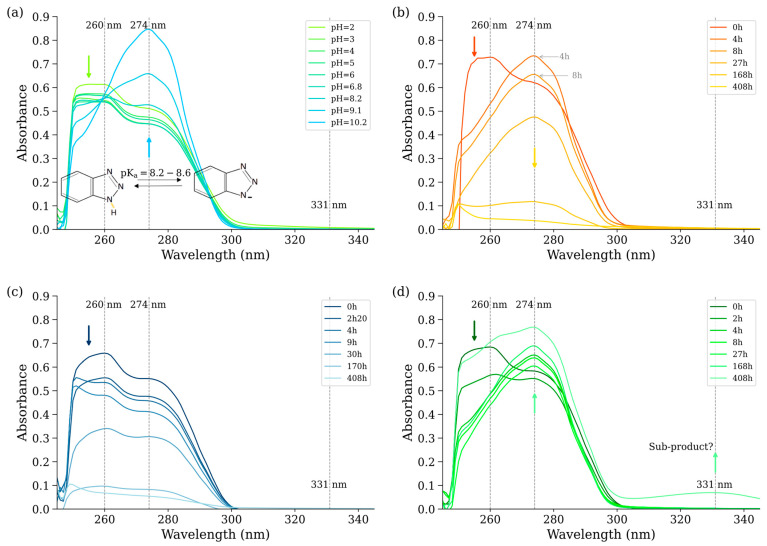
UV-vis absorption spectra of BTA equilibrated in water at various pH levels (**a**), and after reacting with nanomagnetite–biochar composite (**b**), nanomagnetite (**c**), and biochar (**d**). Panels (**b**–**d**) equilibrated at pH7 and with a solid concentration ratio of 2 g/L for B and 15 g/L for M and MB composite were measured at different equilibrium times.

In the kinetic study, the concentration of BTA was monitored using UV–vis spectroscopy at 260 nm for the B and WB experiments (Figure 3c). However, this method proved unsuitable for the M and MB composite experiments due to significant spectral changes over time. Specifically, the UV–vis spectra from these experiments displayed a decrease in absorbance at 260 nm coupled with the increase at 274 nm (Figure 3b,d).

For the MB composite, a continuous absorbance decline across the entire UV–vis spectrum was observed, occurring after 27 h at a solid-to-liquid concentration of 15 g/L and after 17 days at 2 g/L. This behavior allowed for quantitative monitoring of BTA removal using a calibration curve based on absorbance at 274 nm. In contrast, the UV–vis spectra in the M experiments exhibited additional, more complex variations, rendering this method unreliable for accurately tracking BTA concentrations.

Further investigation into the UV–vis spectra of BTA across varying pH levels (Figure 3a) revealed a similar spectral behavior. At pH values up to 8.6 (corresponding to BTA’s pK_a_), a decrease in absorbance at 260 nm was observed alongside an increase at 274 nm, indicative of BTA deprotonation. Below the pKa, under acidic conditions, the spectral changes reversed, with an increase in absorbance at 260 nm and a decrease at 274 nm, reflecting BTA protonation.

In the M- and MB-based experiments, the pH remained constant at close to 7, suggesting that the observed spectral changes are likely attributable to a slow proton exchange process occurring between BTA and the nanomagnetite particle surface. Additionally, in the spectra from the M experiments, a new absorption peak at 331 nm was observed after 17 days, suggesting changes in BTA speciation and the potential formation of a degradation product. In previous studies, multiple sub-products have been reported exclusively in the context of BTA degradation via heterogeneous photochemistry [29]. However, in this study, the formation of this new BTA sub-product occurred under anoxic, dark conditions, implying its linkage to a redox process mediated by interactions with the highly reactive nanomagnetite. As a possible mechanism, we propose that surface protonation of nanomagnetite occurs due to BTA deprotonation. It is well established that protonation of magnetite leads to the release of Fe^2^⁺ ions into the solution (Reaction 1, [30,31]). Subsequently, these Fe^2^⁺ ions may interact with BTA, forming a complex and initiating a redox reaction that reduces BTA.

Fe_3_O_4_ + 2H^+^ → Fe_2_O_3_ + Fe^2+^ + H_2_O (R1)

### 3.5. Kinetic Variations Between B, WB, and MB Materials

**Figure 4:**
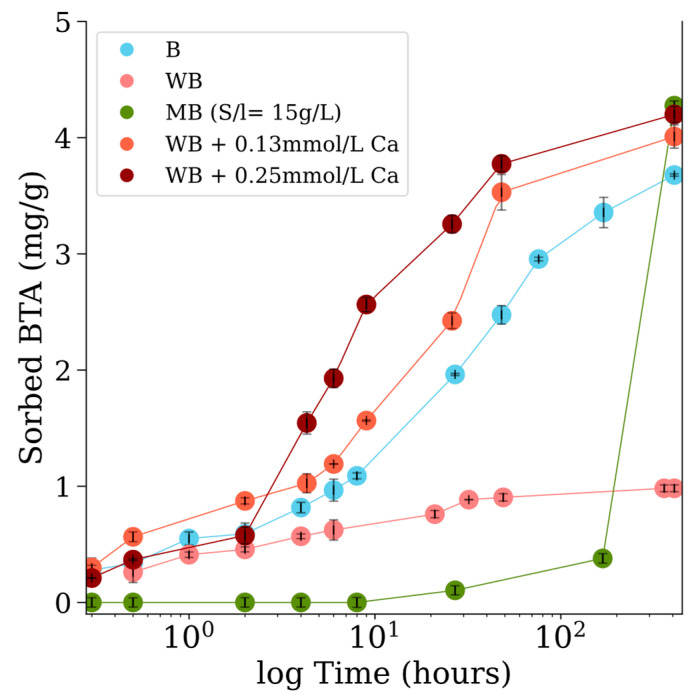
Kinetics BTA adsorption (mg BTA/g adsorbent) for various adsorbent materials: MB—biochar/magnetite (green); B—biochar (blue); WB—biochar washed with HCl (red) materials. Experimental conditions: initial BTA concentration of 100 μmol/L, pH 7, 2 g/L solid concentration. For WB, measurements were taken in presence of 0.13 mmol/L and 0.25 mmol/L Ca^2^⁺. Experiments with MB composite used 15 g/L solid concentrations.

Figure 4 illustrates the results of adsorption experiments designed to assess the efficiency of BTA removal from solution using B, WB, and MB materials. For MB at a solid concentration of 2 g/L, the removal rate was notably slow, with only 3% BTA removal after 17 days. In contrast, at a higher solid concentration of 15 g/L, the removal efficiency increased dramatically, achieving over 96% BTA removal within approximately 17 days (408 h).

Kinetic studies revealed distinct performance differences between B and WB. The acid-washed biochar (WB) demonstrated limited adsorption capability, with only 18% BTA removal after 48 h and 20% after 17 days of equilibrium. Adsorption behavior for WB was well described by both the pseudo-first-order (PFO) and pseudo-second-order (PSO) models (Figure S1). The small sum of squared errors (SSE) values—9083 for the PFO model and 3417 for the PSO model—suggest that the sorption process likely involves a combination of electrostatic interactions and electron donor–acceptor interactions (Table S1).

Biochar surfaces typically exhibit a neutral to negative charge, even under acidic conditions, similar to amphoteric surface groups. Partial protonation of biochar surface groups during acid wash result in a positively charged surface capable of interacting with negatively charged BTA molecules. The proposed mechanism for BTA deprotonation is based on the BTA UV–vis absorption spectra observation. This suggests that the inherent surface properties of biochar may facilitate BTA deprotonation. However, this same characteristic could also contribute to the repulsion of neutral BTA molecules at pH 7, potentially explaining the limited BTA removal efficiency observed in the WB system. Notably, BTA deprotonation was not detected in the UV–vis spectra in biochar experiments, in contrast to observations with nanomagnetite and the nanomagnetite–biochar composite. This discrepancy may be attributed to the rapid nature of the deprotonation and subsequent adsorption reaction, which could occur too quickly to be captured by UV–vis analysis.

The greater BTA removal efficiency observed with B (50% after 48 h and 74% after 17 days equilibrium time) was influenced by the presence of calcite crystals identified within the material structure. Ca^2+^ ions into the solution were release by calcite dissolution with the BTA contaminant, which leads to the BTA deprotonation and the subsequent formation of a ternary complex between Ca^2+^, BTA, and the biochar surface. This is also support with previous studies that also proposed the formation of such ternary complexes as a mechanism for sequestering BTA in soil solutions containing Ca^2+^, given that BTA readily forms complexes with metal ions (Me) [32].

BTA + B^−^ + Me^2+^ → B-Me-BTA (R2)

The influence of Ca^2+^ was further investigated through kinetic experiments using acid-washed biochar with minimal ash ttraces. In these experiments, 0.13 and 0.25 mmol/L of Ca^2+^ were introduced from CaCl₂ powder. These concentrations were chosen based on the observed release of 0.17 mmol/L Ca^2+^ from a 10 g/L biochar suspension, equilibrated at pH 3 for 48 h. We observed that the addition of Ca^2+^ significantly enhanced the removal efficiency of BTA. Specifically, the removal efficiencies were 71% and 75% for 0.13 mmol/L and 0.25 mmol/L Ca^2+^ concentrations, respectively, after 48 h. After 17 days of equilibrium, the efficiencies increased to 80% and 86% for the respective Ca^2+^ concentrations.

### 3.6. Industrial and Environmental Implications

The reactivity of nanomagnetite–biochar composite varies with reaction time, thus with water treatment tank residence time. Within a short time scale (up to 8 h), the removal of BTA by the composite is shown to be due to BTA deprotonation by nanomagnetite surfaces. On a longer time scale, our findings indicate that nanomagnetite iron surface species participate in redox reactions with BTA, as suggested by the new absorption peak at 331 nm observed in the UV–vis spectrum after 17 days. This could be due to a slow degradation of BTA and generation of byproducts. If these byproducts are non-conservative, a simple treatment may allow for the regeneration of the M or MB adsorbent material. To date, as far as we know, BTA degradation has been observed through UV/H₂O₂ treatment, wetlands integrated with solar TiO₂ photocatalytic processes, UV/chlorine processes, and UV-activated peracetic acid (PAA) reactions [29,33]. The resulting byproducts, which appear to be less toxic, stem from hydroxylation, triazole ring opening, and benzene ring cleavage. Additionally, the formation of a ternary surface complex involving BTA, Ca^2^⁺, and the biochar surface suggests that similar mechanisms may occur in other types of biochar. In natural waters and wastewater, cations such as Ca^2^⁺, Mg^2^⁺, and Fe^2^⁺ can influence adsorption, particularly in biochars with low ash content. However, scaling this process to an industrial level presents several challenges and limitations that require further analysis.

One significant challenge is the cost/benefit ratio associated with adsorbent preparation, activation, and regeneration. Although activated biochar with a higher specific surface area (SSA) improves BTA adsorption [34], the specific biochar used in the present study has a relatively low SSA. This likely contributes to the slow adsorption of BTA dependent in part on the Fe^2^⁺ and Ca^2^⁺(=Me^2+^) ions released from nanomagnetite and ash content, respectively, to form biochar–Me–BTA ternary complexes. Industrial-scale biochar production with consistent high SSA may require additional energy input and resource allocation, potentially increasing overall costs. Another important factor is the proportion of nanomagnetite loaded onto the biochar. In this study, we used 50 wt% biochar; increasing the nanomagnetite content could accelerate the degradation and removal of BTA. Fe^2^⁺ release is dependent on the properties of the nanomagnetite, such as size and functionalization. A detailed economic analysis comparing the costs of producing high-SSA biochar (e.g., from steel industry waste) and varying nanomagnetite loads versus the economic benefits of enhanced adsorption efficiency is necessary to justify large-scale implementation.

Long-term stability and consistent performance are also critical concerns. Environmental factors such as pH fluctuations, temperature changes, competing ions, and the overall composition of wastewater could help to degrade the adsorbent or reduce the adsorbent effectiveness [13]. For instance, acidic water may increase the solubility of nanomagnetite, leading to larger Fe^2^⁺ release, but could also induce nanomagnetite oxidation to maghemite according to Reaction (1). It remains unclear whether maghemite would improve or hinder BTA removal, and this requires further investigation. On the other hand, the synergistic or antagonistic effects of wastewater matrix components on BTA adsorption is important for optimizing composite formulations for diverse wastewater compositions. To address this point, long-term (at least six months) kinetic studies are necessary to evaluate the durability and adsorption capacity of biochar, nanomagnetite, maghemite, and nanomagnetite–biochar composites under simulated field conditions. Further investigations are also needed to gain deeper insights into the roles of Fe^2^⁺ and Mg^2^⁺ in enhancing the performance of B and MB composites for industrial applications.

Regenerative capabilities represent another aspect of industrial scalability. While preliminary evidence suggests that non-conservative byproducts may enable straightforward regeneration of the adsorbent material, repeated regeneration cycles could lead to structural degradation or reduced adsorption efficiency, as evidenced in other biochar studies [35]. Future studies should focus on characterizing the degradation pathways of BTA induced by nanomagnetite or Fe^2^⁺ in solution. Investigations into the mechanical and chemical resilience of B or MB composite during multiple regeneration cycles will provide valuable insights into their practical lifespan and replacement frequency.

Ultimately, while the present preliminary results are promising, comprehensive studies addressing these economic, stability, and regenerative challenges are indispensable to facilitate the transition from laboratory-scale experiments to robust, cost-effective industrial applications. This study, along with future investigations, underscores the importance of the wastewater treatment plant (WWTP) matrix and Me^2+^ concentration in determining sorption performance and highlights the need for holistic evaluations encompassing technical, environmental, and economic considerations.

## 4. Conclusions

In conclusion, this study evaluated the immobilization of benzotriazole (BTA), an emerging contaminant, using three adsorbents: commercial biochar (B), nanomagnetite (M), and nanomagnetite–biochar composite (MB). Nanomagnetite showed potential for BTA oxidation after 2 weeks, and MB demonstrated a slow but almost complete BTA adsorption over time. Both nanomagnetite and MB composite induced proton transfer from BTA to M surface in the beginning of the reaction, as evidenced by UV–vis spectra. The slow dissolution of M at a neutral pH may induce an Fe^2+^ release and consequent B-Me^2+^-BTA complex similar to what is observed for Me^2+^ = Ca^2+^. Indeed, while BTA removal efficiency was over 75% within 48 h and exceeding 86% after two weeks with the addition of 0.25 mmol Ca^2+^, the primary removal mechanism involves the formation of B-Me^2+^-BTA ternary surface complexes between BTA, Ca^2+^, and the biochar surface. This work advances the development of low-cost, environmentally friendly filtration materials for potential use in wastewater treatment.

## Data Availability

The data that support the findings of this study are included in the paper.

## Supplementary Materials

The following supporting information can be downloaded at: https://www.mdpi.com/article/10.3390/nano15020115/s1, Figure S1: Non-linear pseudo-first-order (PFO) and pseudo-second-order (PSO) kinetic models for the adsorption of BTA onto different biochar types: B (biochar), WB (biochar washed with HCl), and WB supplemented with 0.13 mmol/L and 0.25 mmol/L Ca(II). Table S1: Isotherm modeling results related to the BTA adsorption onto biochar and acid-washed biochar enhanced with calcium.
