# Supplementary material for: Evaluation of Nanomagnetite–Biochar Composite for BTA Removal
Source: Nanomaterials (Basel). 2025 Jan 14;15(2):115. doi: 10.3390/nano15020115 (PMC11767809; doi:10.3390/nano15020115)
Supplement: Supplementary file 1 [file nanomaterials-15-00115-s001.zip › nanomaterials-3412713-supplementary.pdf]

Supplementary Material

Evaluation of Nanomagnetite–Biochar Composite for BTA Removal

Carolina Guida[a], Nathaniel Findling[a], Valerie Magnin[a], Fabienne Favre Boivin[b], Laurent Charlet[a*]

[a]ISTerre, University Grenoble Alpes, University Savoie Mont Blanc, CNRS, IRD, University Gustave Eiffel,

38058 Grenoble, France; carolina.guida.m@gmail.com (C.G.); nathaniel.findling@universite-paris-saclay.fr (N.F.); valerie.magnin@univ-grenoble-alpes.fr (V.M.); charlet38@gmail.com (L. C.)

[b]Institute des Technologies de l'Environnement Construit, University of Applied Sciences and Arts Western Switzerland, 1700 Fribourg, Switzerland; fabienne.favre@hefr.ch

[*]Corresponding author. E-mail: charlet38@gmail.com

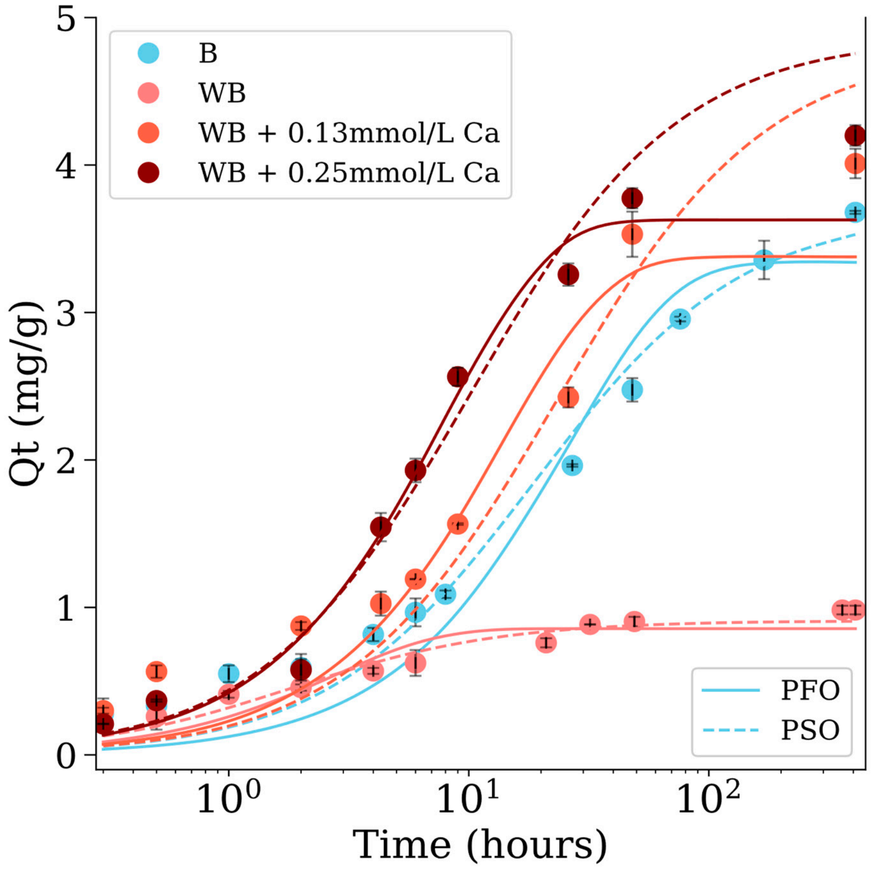

Figure S1. Nonlinear pseudo-first-order (PFO) and pseudo-second-order (PSO) kinetic models for the adsorption of BTA onto different biochar types: B (biochar), WB (biochar washed with HCl), and WB supplemented with 0.13 mmol/L and 0.25 mmol/L Ca^{2+}. Experimental conditions: initial BTA concentration of 100 μmol/L, pH 7.0, and solid concentration of 2 g/L.

Table S1. Isotherm modeling results related to the BTA adsorption onto biochar and acid-washed biochar enhanced with calcium.

	Pseudo First Order				Pseudo Second Order			
Adsorbent material	q_e (mg/g)	k_1 (h^{-1})	SSE	$t_{1/2}$ (h)	q_e (mg/g)	k_2 (h^{-1})	H_0 (mg/(g*h))	SSE
B	3.34	0.038	0.97	18.3	3.69	0.014	0.197	0.43
WB	0.86	0.360	0.09	1.9	0.91	0.596	0.493	0.03
WB+0.13 mmol/L Ca(II)	3.38	0.071	0.65	9.7	4.80	0.009	0.207	0.94
WB+0.25 mmol/L Ca(II)	3.63	0.125	0.16	5.6	4.87	0.020	0.485	0.35
